# Where is quality in health systems policy? An analysis of global policy documents

**DOI:** 10.1016/S2214-109X(18)30375-9

**Published:** 2018-11-01

**Authors:** Keely Jordan, Robert Marten, Oye Gureje, Bernadette Daelmans, Margaret E Kruk

**Affiliations:** * NYU College of Global Public Health, New York University, New York, NY, USA (KJ); London School of Hygiene & Tropical Medicine, London, UK (RM); Department of Psychiatry, University of Ibadan, Ibadan, Nigeria (OG); Department of Maternal, Newborn, Child and Adolescent Health, World Health Organization, Geneva, Switzerland (BD); and Department of Global Health and Population, Harvard T H Chan School of Public Health, Boston, MA, USA

Addressing the quality of care provided by health systems is necessary if countries hope to improve health outcomes following the adoption of universal health coverage (UHC) as a global priority.^1^ The Millennium Development Goals (MDGs) spurred expansion of funding for health and an increase in coverage of services.^2^ However, the breadth of global health ambition within the Sustainable Development Goals (SDG) demands that policymakers move beyond coverage to understand what happens once people reach a health facility or come into contact with health services.^3^ The Lancet Global Health Commission on High Quality Health Systems in the SDG Era^4^ defines high-quality health systems as those that “optimise health in a given context by consistently delivering care that improves or maintains health, being valued and trusted by all people, and responding to changing population needs”. The Commission also proposes new ways to define, measure, and improve the performance of health systems. It states that the quality of health systems should be primarily measured in terms of the processes of care and health impacts rather than inputs such as staff and equipment. We aimed to explore how the concept of health system quality has been considered in key health systems frameworks and policies since 2000.

We searched the WHO website for World Health Reports, frameworks, and reports that included “health system” in the title or had a key aim mentioning health systems. We restricted our focus to WHO as it is the UN’s leading technical agency for health, it is focused on health systems, and it has a powerful role in shaping the normative paradigms in the health systems discourse. We also included documents from the Alliance for Health Policy and Systems Research—an international partnership hosted by WHO that promotes the use of health policy and systems research to strengthen health systems in low-income and middle-income countries (LMICs). For Alliance reports, we included the flagship reports, which according to their mission are aimed at “stimulating thinking and debate on emerging gaps and challenges in strengthening health systems”. Given the close link between UHC and health-system quality, we also included the 2015 and 2017 UHC monitoring reports. We did not include condition-specific documents.

We manually searched each document for its use and discussion of “quality”. Where quality was not explicitly mentioned, we searched for other health system concepts, such as “service access” and “coverage”. The content was then summarised and analysed for common themes. We also compared the content to the Commission’s definition and framework.

We retrieved 20 documents: 13 from WHO and seven from the Alliance (table). Five documents did not explicitly discuss quality as defined by the Commission (*2006 World Health Report, 2007 Sound Choices Enhancing capacity for evidence-informed health policy, 2012 WHO Strategy on research for health, 2012 Changing mindsets WHO strategy on health policy and systems research, and the 2016 Open mindsets participatory leadership for health*). Among those that did mention quality, three themes emerged. The first was that documents overall emphasised access to health services; more recent ones included the equity of access. The World Health Report 2000^5^ presented a framework for understanding health systems and clearly placed LMIC health system performance on the political and research agendas. The report briefly discussed quality care for the vulnerable, who receive the “worst levels of responsiveness—they are treated with less respect for their dignity, given less choice of service providers and offered lower-quality amenities”. Shortly thereafter, the Alliance biennial report *Strengthening health systems: the role and promise of health policy and systems research* (2004) highlighted inequalities in access to care.^6^ In 2008 The World Health Report addressed the role that primary health care could play in getting health development “back on track”.^7^ The report found a wide-range of inequities in health outcomes, access to care, and payment for services and warned that “left to their own devices, health systems do not move towards greater equity.”

**Table:** Health system policy documents retrieved and analysed

	Source	Title	Significance
2000	WHO	World Health Report 2000–Health systems: improving performance	One of the first conceptual frameworks for health systems
2004	Alliance	Strengthening health systems: the role and promise of health policy and systems research	First of a series of Alliance biennial reports
2005	WHO	World Health Report 2005–Make every mother and child count	Addresses the need to provide families universal access to a continuum of care
2006	WHO	World Health Report 2006–Working together for health	Assessment of workforce crisis in global health
2007	WHO	Strengthening health systems to improve health outcomes	Discussion of building blocks that make up health systems and how to strengthen them
2007	Alliance	Sound choices: enhancing capacity for evidence-informed health policy	Looks at the capacity to generate and apply research evidence
2008	WHO	World Health Report 2008–Primary health care: now more than ever	The role of primary health care in health systems responding to changing challenges
2008	WHO	Scaling up research and learning for health systems	WHO High-level Consultation and Task Force Report on acceleration of the development of research tools and common frameworks for health systems strengthening
2009	Alliance	Systems thinking for health systems strengthening	Connected HPSR to complex adaptive systems
2010	WHO	World Health Report 2010–Health Systems Financing: The path to universal coverage	The role of universal health coverage in addressing current health challenges
2012	WHO	WHO Strategy on Research for Health	Prioritised research that met health needs and underscored investments in capacity development and knowledge translation
2012	Alliance	Changing mindsets: WHO strategy on health policy and systems research	First WHO Strategy focused on HPSR
2013	WHO	World Health Report 2013–Research for universal health coverage	Calls for greater investment in HPSR in LMICs
2014	Alliance	Medicines in health systems: advancing access, affordability and appropriate use	Addresses the challenges to achieve equitable access to essential medicines
2015	WHO	Tracking universal health coverage: first global monitoring report	WHO and World Bank joint monitoring report on global assessment of the current state of universal health coverage
2016	WHO	WHO framework on integrated, people-centred health services	Considers patient rights across all levels of the health system
2016	Alliance	Open mindsets: participatory leadership for health	Assesses the different aspects of leadership within the health system
2016	WHO	Global strategy on human resources for health: workforce 2030	Examines the role of health workers within health system strengthening on the path to universal health coverage
2017	Alliance	World report on health policy and systems research	First world report aimed at monitoring and measuring developments in HPSR
2017	WHO	Tracking universal health coverage: 2017 global monitoring report	WHO and World Bank follow-up monitoring report on goals and aims of universal health coverage

HPSR=health policy and systems research. LMIC=low-income and middle-income country.

The World Health Report 2013: research for universal health coverage^8^ expanded the definition of equity and noted that achieving universal health coverage means “reaching remote and conflict-affected populations where the challenges of dilapidated infrastructure and the shortage of qualified, skilled human resources are enormous”. Even though the report recognised that it is not just the quantity of services that is important but also their quality, the majority of quality improvement suggestions it proposed focused on system inputs rather than process of care or health impacts. The 2015 and 2017 *Tracking universal health coverage* reports^1,9^ recognised that “ensuring access to quality health services is a central tenet of UHC”. Both reports highlighted the role that quality plays in effective coverage (health services obtained in a timely manner and at a level of quality necessary to obtain the desired effect and potential health gains) but noted the challenge in coming to an agreed definition of quality and in monitoring it effectively.

The second theme to emerge was that what the Commission calls “foundations” (particularly workforce and tools) have been the focus of discussions on quality in the reviewed documents. This dimension of care includes the factors affecting the context in which care is delivered. It does not, however, address the process or impacts of care. Prior to 2007, the reports reviewed mentioned quality improvement with regard to better infrastructure and a prepared workforce.^5,10^ In 2007, WHO published *Framework for action: strengthening health systems to improve health outcomes*,^11^ which elaborated the building blocks of a well functioning health system and which became the basis of measuring health system performance. The building blocks are: health services; health workforce; health information; medical products, vaccines, and technologies; health financing; and leadership and governance.^23^ Quality and access were featured as the outputs of such a system. The report suggested that the building blocks should be used to monitor trends in health systems and performance. It does not specify, however, how the building blocks could come together to produce quality and eventually better health, with the assumption being that good service quality flows naturally from having the building blocks in place.

Other health documents applied the building blocks for understanding and analysing health systems. For example, the Alliance report *Sound choices: enhancing capacity for evidence-informed health policy*,^12^ the WHO High-level Consultation and Task Force Report *Scaling up research and learning for health systems*,^13^ and the 2009 Alliance report *Systems thinking for health systems strengthening*^14^ focused their quality measures on the building blocks. *Sound choices*^12^ noted that health policy and systems research should aim to produce “reliable and rigorous evidence” on any or all of the six building blocks. The World Health Report 201015 emphasised the need to analyse one of the building blocks—financing mechanisms—to “enhance the quality and quantity of service delivery, and ensure appropriate medicines and technologies are available”.

The third theme was the people-centred care is emerging as a focus. The World Health Report 2000^5^ included responsiveness—and, within that, respect for persons—as a key component of health system performance, and the World Health Report 2008^7^ noted that “people-centredness” becomes the “clinical method of participatory democracy”, measurably improving the quality of care, the success of treatment, and the quality of life of those benefiting from such care”. The World Health Report 2013^8^ echoed this call for “integrated, high-quality, patient-centred services at all levels of primary to tertiary care”. The inclusion of patient-centred services in these reports helped solidify patient-centred care in the quality discussion.

In 2016, the WHO *Framework on integrated, people-centred health services*^16^ addressed people’s needs and preferences across all levels of the health system. The report points out that “where it is accessible, care is too often fragmented or of poor quality, and consequently the responsiveness of the health system and satisfaction with health services remain low in many countries”. The report marks a progression in global health policy from the vertical to the horizontal and from the technocratic to the user-focused, noting that “hospital-based, disease-based and self-contained ‘silo’ curative care models further undermine the ability of health systems to provide universal, equitable, high-quality and financially sustainable care”. On the path to UHC, the framework suggests a health system in which “all people have equal access to quality health services that are co-produced in a way that meets their life course needs, are coordinated across the continuum of care, and are comprehensive, safe, effective, timely, efficient and acceptable; and all careers are motivated, skilled and operate in a supportive environment”. Empowering and engaging users and providers are core values of the framework and the WHO calls for their implementation to be country-led, equity-focused, participatory, systems strengthening, evidence based, results-oriented, ethics-based, and sustainable.

The Alliance report *Open mindsets: participatory leadership for health* also recognised the importance of quality leadership to “raise the performance of the health system high above the welter of complexity”.^17^ The same year, the WHO *Global strategy on human resources for health: workforce 2030*^18^ recognised that it is not just the availability of health workers that is needed but a workforce that is “motivated to deliver quality care and build a positive relationship with the patients”. These values were again seen in the 2017 *World report on health policy and systems research*^19^ and the Alliance’s call for an interdisciplinary approach to improving user quality with a focus on user experience. The 2017 *Tracking universal health coverage report*^9^ included responsiveness or people-centredness within its dimensions of quality and noted the importance but also difficulty in properly measuring these elements of quality.

This Comment presents an overview of the role that quality has played in health system policy as elaborated by the WHO and the Alliance for Health Policy and Systems Research since 2000. Our findings pertain to this set of reports. We did not review the many disease-specific and other more narrowly focused documents that address quality of care—eg, tuberculosis, HIV, and maternal health reports or policies on patient safety or antimicrobial resistance. There are also many documents written by agencies other than WHO, such as the World Bank, UNICEF, and UNFPA. This would be an area of future inquiry for health system scholars.

As countries continue to move towards UHC and implement the broader SDG3 agenda, WHO and other global actors will need to raise the profile of health system quality in their support to countries and in normative guidance. This will require reorienting health systems as primarily accountable to people, raising our expectation of what quality of services health systems provide to people, and focusing health systems measurement on processes and outcomes of care that are most valued by people. It will also require compiling an evidence base of what works to improve health system quality at scale to meet the increasingly complex and pressing needs of populations in LMICs.

The authors alone are responsible for the views expressed in this article and they do not necessarily represent the views, decisions, or policies of the institutions with which they are affiliated. We declare no competing interests.
